# Significant Decline in HCV‐Related Mortality From Liver Cirrhosis and Chronic Liver Disease (2015–2022): A Sentinel Center Study

**DOI:** 10.1111/liv.70388

**Published:** 2025-10-07

**Authors:** Cheng‐Yeh Yang, Chih‐Yun Lin, Jing‐Houng Wang, Chun‐Yen Lin, Rong‐Nan Chien, Sheng‐Nan Lu

**Affiliations:** ^1^ Division of Hepato‐Gastroenterology, Department of Internal Medicine Kaohsiung Chang Gung Memorial Hospital Kaohsiung Taiwan; ^2^ Biostatistics Center of Kaohsiung Chang Gung Memorial Hospital Kaohsiung Taiwan; ^3^ College of Medicine Chang Gung University Taoyuan Taiwan; ^4^ Division of Hepato‐Gastroenterology Linkou Chang Gung Memorial Hospital Taoyuan Taiwan

**Keywords:** chronic liver disease, hepatitis C, liver cirrhosis, mortality, secular trend

## Abstract

**Background:**

Reducing mortality attributable to hepatitis C virus (HCV) is a key target in the World Health Organization (WHO) HCV elimination strategy. While liver cancer registration in Taiwan includes hepatitis C antibody (anti‐HCV) status, no national registry records viral etiologies for deaths from liver cirrhosis and chronic liver disease (LC/CLD). Therefore, this sentinel study aimed to estimate national trends in HCV‐related LC/CLD mortality.

**Methods:**

The study period spanned from 2015 to 2022. According to ICD‐10 codes K70, K73 and K74, the annual mortality numbers for LC/CLD were extracted from national biostatistics. Using the Chang Gung Research Database (CGRD), cases that died from LC/CLD with available results for both HBsAg and anti‐HCV tests were included in the analysis. Based on the proportions of HCV distributions from CGRD, the national viral etiology‐specific case numbers were estimated using a direct standardised method.

**Results:**

A total of 35 413 patients died from LC/CLD nationally, with 15 445 of them having medical records in CGRD, and 5940 (16.8%) having results for both HBsAg and anti‐HCV tests. The national case number was 4878 in 2015, decreasing to 4107 (−15.8%) in 2022. The proportion of anti‐HCV decreased from 25.7% to 16.6% (−36.2%). Direct standardisation showed that the estimated national case number for anti‐HCV decreased from 1424 to 811 (−43%).

**Conclusions:**

From 2015 to 2022, Taiwan experienced a 43% decline in HCV‐related LC/CLD mortality, supporting progress toward national HCV elimination goals.

**Summary**

- This study found that deaths from hepatitis C virus (HCV)‐related liver cirrhosis and chronic liver disease have declined significantly in Taiwan since 2015.
- The results show that national treatment programs can rapidly reduce liver‐related deaths, demonstrating the impact of public health policies on saving lives.

**Abbreviations**
Anti‐HCV: hepatitis C antibody
B: HBsAg‐positive and anti‐HCV‐negative patients
BC: HBsAg‐ and anti‐HCV‐positive patients
C: HBsAg‐negative and anti‐HCV‐positive patients
CGMH: Chang Gung Memorial Hospital
CGRD: Chang Gung Research Database
DAAs: direct‐acting antivirals
HBsAg: hepatitis B surface antigen
HCC: hepatocellular carcinoma
HCV: hepatitis C virus
HPA: Health Promotion Administration
ICD‐10: International Classification of Diseases, 10th Revision
IFN‐α: interferon alpha
LC/CLD: liver cirrhosis and chronic liver diseases
MASLD: metabolic dysfunction‐associated steatotic liver disease
NBC: HBsAg‐ and anti‐HCV‐negative patients
NHI: National Health Insurance
WHO: World Health Organization

## 1 Introduction

Hepatitis C virus (HCV) infection is one of the leading causes of liver diseases worldwide [1, 2]. Although an effective vaccine is unavailable, the development of direct‐acting antivirals (DAAs) has enabled HCV elimination [3, 4]. By 2020, the global prevalence of HCV infection had declined to 0.7% [5]. However, a large‐scale study estimated the prevalence of HCV infection in Taiwan to be 4.4%, with notable geographic and generational clustering [6]. These findings underscore the necessity of targeted HCV prevention and control strategies in Taiwan.

In 2016, the World Health Organization (WHO) set a goal to eliminate global viral hepatitis by 2030, aiming to reduce HCV incidence by 90% and mortality by 65% [7, 8]. In 2023, the WHO further elaborated on specific strategies to achieve these ‘impact targets’, highlighting the challenge of obtaining comprehensive nationwide data. In such cases, the WHO recommends the use of subnational sentinel center data as an alternative approach for country‐level validation [9].

The Taiwanese government is committed to achieving HCV elimination ahead of the 2030 WHO target by implementing a series of strategic policies [10]. To facilitate early detection, a nationwide screening program was launched in 2011 and expanded to include individuals aged 45 to 79 years in 2020 [11, 12]. Concurrently, Taiwan introduced conditional DAA reimbursement in 2017, followed by full, unconditional reimbursement in 2020 [13, 14, 15]. These initiatives have established a robust foundation for the national HCV elimination program [16].

Similar to many other countries, Taiwan collects nationwide hospital‐based data through the Taiwan Cancer Registry, which includes hepatitis B surface antigen (HBsAg) and hepatitis C antibody (anti‐HCV) testing for all liver cancer patients [17, 18]. However, an analysis of Taiwan's national mortality database revealed a lack of comprehensive HBsAg and anti‐HCV testing data for patients whose cause of death was attributed to liver cirrhosis and chronic liver disease (LC/CLD). This data gap impedes a thorough understanding of the role of HCV in non‐cancerous liver‐related mortality.

Notably, the Chang Gung Memorial Hospital (CGMH) healthcare system is the largest in Taiwan, with the highest number of inpatient beds and annual patient volume. The affiliated Chang Gung Research Database (CGRD) is the largest multi‐institutional electronic medical records database in Taiwan. It has been widely used in evidence‐based studies, and its data accuracy has been validated in numerous research publications [19, 20, 21].

In this study, we aimed to address the data gap described above by analysing secular trends in HCV‐related LC/CLD mortality using data from the CGRD and national mortality databases. By providing comprehensive insights into the role of HCV in LC/CLD mortality, this research contributes to understanding the epidemiological impact of HCV elimination efforts in Taiwan.

## 2 Materials and Methods

### 2.1 Patients and Methods

**FIGURE 1:**
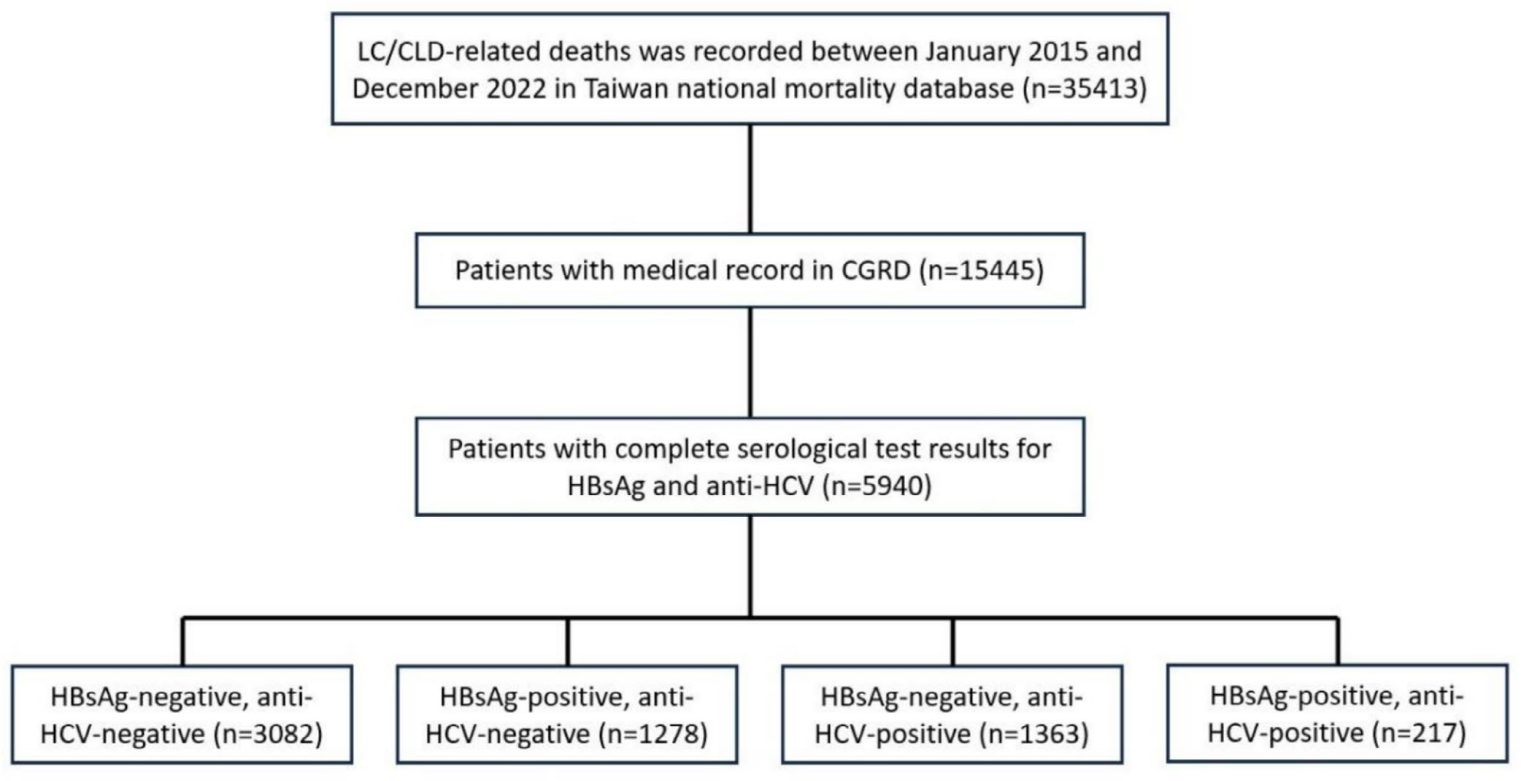
Flowchart of study population and grouping. CGRD, Chang Gung Research Database; LC/CLD, liver cirrhosis and chronic liver disease.

A flowchart of the study population and grouping is shown (Figure 1). This retrospective study utilised mortality data from Taiwan's national mortality database to analyse trends from January 2015 to December 2022. Annual mortality counts were extracted, and cases with at least one of the following International Classification of Diseases, 10th Revision (ICD‐10) codes—K70 (alcoholic liver disease), K73 (chronic hepatitis, not elsewhere classified), and K74 (fibrosis and cirrhosis of the liver)—were identified as LC/CLD‐related deaths. To minimise potential overlap with hepatocellular carcinoma (HCC), individuals with HCC (C22.0) recorded as either the underlying or an associated cause of death were excluded from the analysis.

To estimate the distribution of viral hepatitis etiology among LC/CLD‐related deaths, data were further retrieved from the CGRD, which encompasses medical records from all CGMH branches across northern, central and southern Taiwan. CGRD was utilised as a representative sentinel center system, providing detailed clinical and laboratory data. To ensure accuracy, duplicate records across hospital branches were removed. Cases meeting the LC/CLD mortality criteria and with complete serological test results for HBsAg and anti‐HCV were included in the analysis.

Mortality trends were analysed by stratifying cases according to the year of death, HBsAg status and anti‐HCV status. Patients were categorised into four groups based on serological test results: HBsAg‐negative, anti‐HCV‐negative; HBsAg‐positive, anti‐HCV‐negative; HBsAg‐negative, anti‐HCV‐positive; and HBsAg‐positive, anti‐HCV‐positive. A patient was considered positive for HBsAg or anti‐HCV if there was any documented positive result at any time during the observation period; repeated testing or subsequent negative results did not alter classification.

### 2.2 Statistical Analysis

The proportions derived from CGRD data were applied to national mortality statistics via direct standardisation to estimate the annual mortality burden attributable to HBsAg and HCV in Taiwan. Descriptive statistics were used to summarise the annual number of LC/CLD‐related deaths and mortality rates. Linear trend analysis was performed using the Cochran‐Armitage trend test to assess changes over time, while the chi‐square test was used to compare categorical variables. Statistical significance was set at a *p*‐value < 0.05. To estimate nationwide trends in HBsAg‐ and HCV‐related LC/CLD mortality, CGRD data were extrapolated using statistical adjustment factors derived from national mortality records. All statistical analyses were conducted using the SAS Enterprise Guide 8.2.

### 2.3 Ethics Statement

This study was approved by the Institutional Review Board of Chang Gung Memorial Hospital (IRB No. 202401570B0) and conducted in accordance with the principles of the Declaration of Helsinki and the International Conference on Harmonisation for Good Clinical Practice.

## 3 Results

### 3.1 Statistics of LC/CLD‐Related Mortality From the National Database and CGRD

Data from the Taiwan national mortality database were analysed to summarise the annual mid‐year population, LC/CLD‐related deaths, and mortality rate per 100 000 population from 2015 to 2022 (Table 1). A consistent decline in both the absolute number of LC/CLD‐related deaths and the corresponding mortality rate was observed throughout the study period (*p* value for linear trend < 0.001). The total number of LC/CLD‐related deaths decreased from 4878 in 2015 to 4107 in 2022. Notably, the LC/CLD mortality rate in 2022 was reduced by 15.8% compared with that in 2015.

**TABLE 1:** Liver cirrhosis and chronic liver disease‐related mortality from the Taiwan national database and CGRD.

Year	Mid‐year population	Mortality statistics of liver cirrhosis and chronic liver disease				
		Total number of mortality cases nationwide	National mortality rate (/100 000)	Mortality reduction (%) (compared to 2015)	Total number of mortality cases from CGRD	Proportion of CGRD cases nationwide (%)
2015	23 462 914	4878	20.8	Reference year	2090	42.8%
2016	23 515 945	4930	21.0	1.1%	2099	42.6%
2017	23 555 522	4739	20.1	−2.8%	2003	42.3%
2018	23 580 080	4490	19.0	−8.0%	1965	43.8%
2019	23 596 027	4240	18.0	−13.1%	1866	44.0%
2020	23 582 179	3964	16.8	−18.7%	1769	44.6%
2021	23 468 275	4065	17.3	−16.7%	1795	44.2%
2022	23 319 977	4107	17.6	−15.8%	1858	45.2%
*p* for trend	—	—	< 0.001	—	—	—

Abbreviation: CGRD, Chang Gung Research Database.

Additionally, data from the CGRD were analysed after removing duplicate patient records across multiple hospital branches. The number of LC/CLD‐related deaths recorded at CGMH from 2015 to 2022 is also presented (Table 1). LC/CLD‐related deaths recorded at CGMH consistently accounted for over 40% of all nationwide LC/CLD‐related deaths each year. From January 2015 to December 2022, a total of 35 413 LC/CLD‐related deaths were reported nationwide, among which 15 445 cases were recorded at CGMH, representing 43.6% of the national total.

### 3.2 Distribution of LC/CLD‐Related Mortality by HBsAg and Anti‐HCV Status in CGRD

**FIGURE 2:**
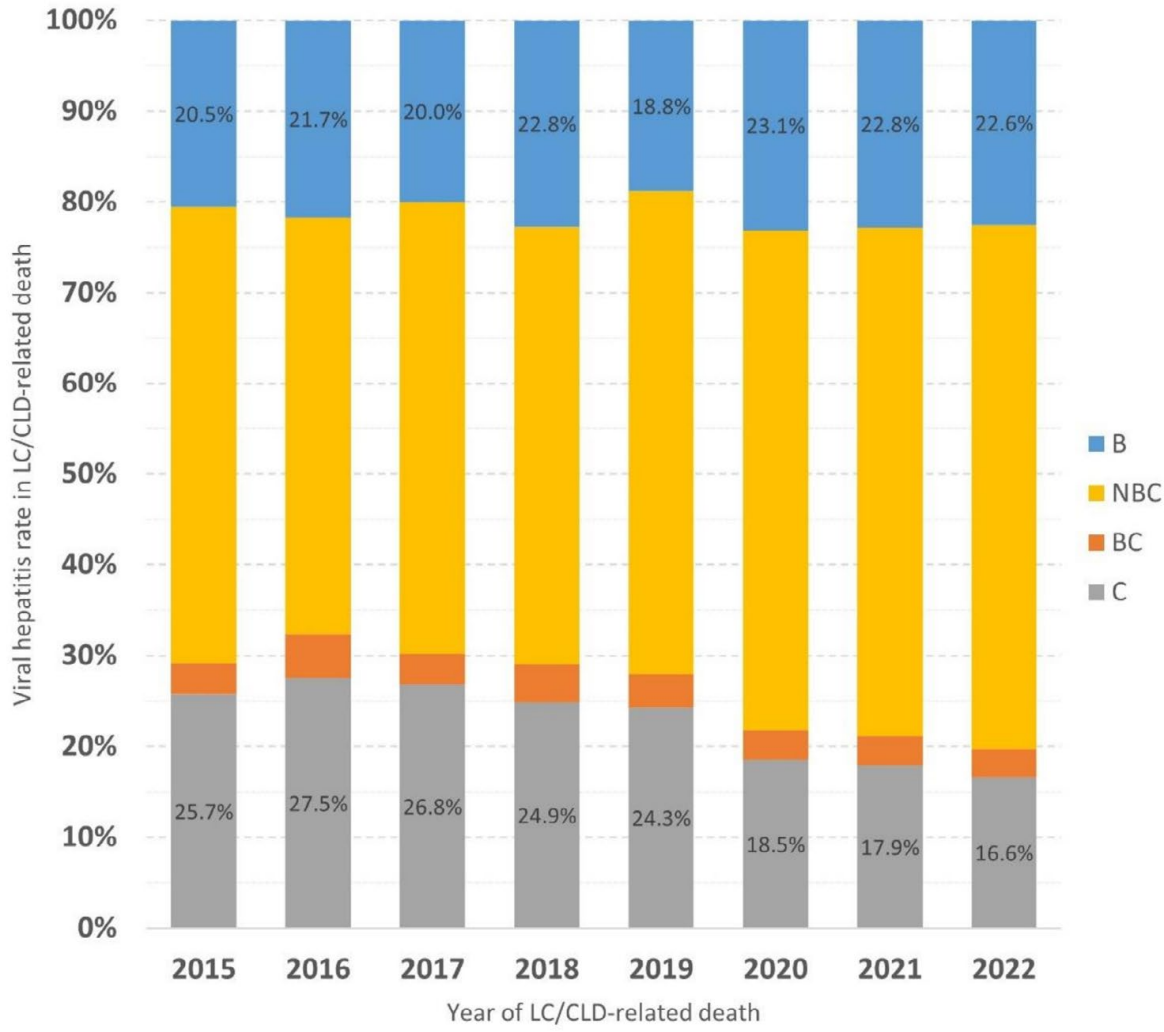
Secular trends of LC/CLD‐related mortality based on viral etiology in CGRD. B, HBsAg‐positive and anti‐HCV‐negative patients; BC, HBsAg‐ and anti‐HCV‐positive patients; C, HBsAg‐negative and anti‐HCV‐positive patients; LC/CLD, liver cirrhosis and chronic liver disease; NBC, HBsAg‐ and anti‐HCV‐negative patients.

Among the 15 445 LC/CLD‐related deaths recorded at the CGMH, patients with complete serological data for both HBsAg and anti‐HCV were identified, yielding a final cohort of 5940 patients. These cases accounted for 16.8% of all LC/CLD‐related deaths nationwide. Patients were further stratified by year and serological test results for HBsAg and anti‐HCV (Table 2 and Figure 2).

**TABLE 2:** Distribution of LC/CLD‐related mortality by HBsAg and anti‐HCV status in CGRD.

		2015	2016	2017	2018	2019	2020	2021	2022	*p* for trend	Total
B	Count	154	171	161	173	134	156	162	167	—	1278
	% within year	20.5%	21.7%	20.0%	22.8%	18.8%	23.1%	22.8%	22.6%	0.203	21.5%
BC	Count	26	38	27	32	26	22	23	23	—	217
	% within year	3.5%	4.8%	3.4%	4.2%	3.7%	3.3%	3.2%	3.1%	0.205	3.7%
C	Count	193	217	216	189	173	125	127	123	—	1363
	% within year	25.7%	27.5%	26.8%	24.9%	24.3%	18.5%	17.9%	16.6%	< 0.001	22.9%
NBC	Count	377	362	401	366	379	372	398	427	—	3082
	% within year	50.3%	45.9%	49.8%	48.2%	53.2%	55.1%	56.1%	57.7%	< 0.001	51.9%
Total	Count	750	788	805	760	712	675	710	740	—	5940
	% within year	100.0%	100.0%	100.0%	100.0%	100.0%	100.0%	100.0%	100.0%	—	100.0%

Abbreviations: B, HBsAg‐positive and anti‐HCV‐negative patients; BC, HBsAg‐ and anti‐HCV‐positive patients; C, HBsAg‐negative and anti‐HCV‐positive patients; NBC, HBsAg‐ and anti‐HCV‐negative patients.

The analysis revealed that 3082 patients (51.9%) were HBsAg‐negative and anti‐HCV‐negative, representing the largest subgroup. The annual proportion of these patients ranged from 45.9% to 57.7%, showing a significant increasing trend over time (*p* value for linear trend < 0.001). Meanwhile, 1278 patients (21.5%) were HBsAg‐positive and anti‐HCV‐negative, with an annual distribution ranging from 18.8% to 23.1%, showing no apparent trend over time (*p* value for linear trend = 0.203). In contrast, 1363 patients (22.9%) were HBsAg‐negative and anti‐HCV‐positive. A decreasing trend was observed in this group, with its proportion declining from 25.7% in 2015 to 16.6% in 2022, representing a 36.2% reduction relative to the 2015 baseline (*p* value for linear trend < 0.001).

### 3.3 Linear Trend Analysis of HCV‐Related LC/CLD Mortality in CGRD

**FIGURE 3:**
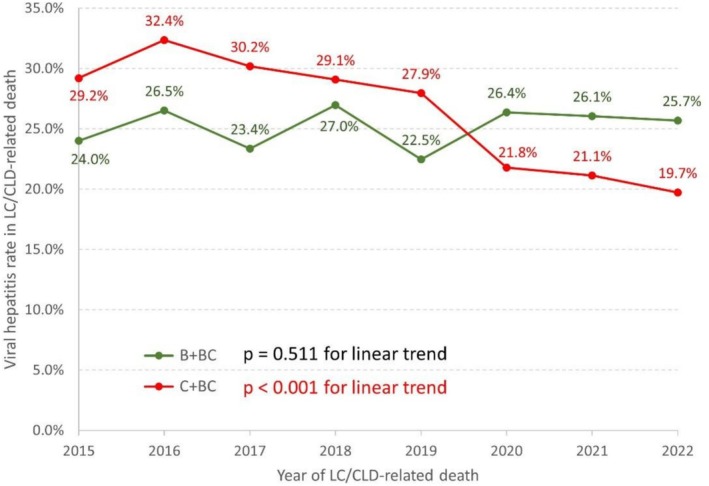
Linear trend analysis of LC/CLD‐related mortality in CGRD showed a significant decline among anti‐HCV‐positive patients, regardless of HBsAg status (*p* for trend < 0.001). However, no significant change was observed among HBsAg‐positive patients, regardless of anti‐HCV status (*p* for trend = 0.511). B, HBsAg‐positive and anti‐HCV‐negative patients; BC, HBsAg‐negative and anti‐HCV‐positive patients; C, HBsAg‐negative and anti‐HCV‐positive patients; LC/CLD, liver cirrhosis and chronic liver disease.

A linear trend analysis was conducted, revealing no statistically significant change in the annual proportion of HBsAg‐positive patients, regardless of anti‐HCV status (*p* value for linear trend = 0.511). However, a significant downward trend was identified among anti‐HCV‐positive patients, irrespective of HBsAg status (*p* value for linear trend < 0.001) (Figure 3).

### 3.4 Secular Trends in Nationwide HCV‐Related LC/CLD Mortality

**FIGURE 4:**
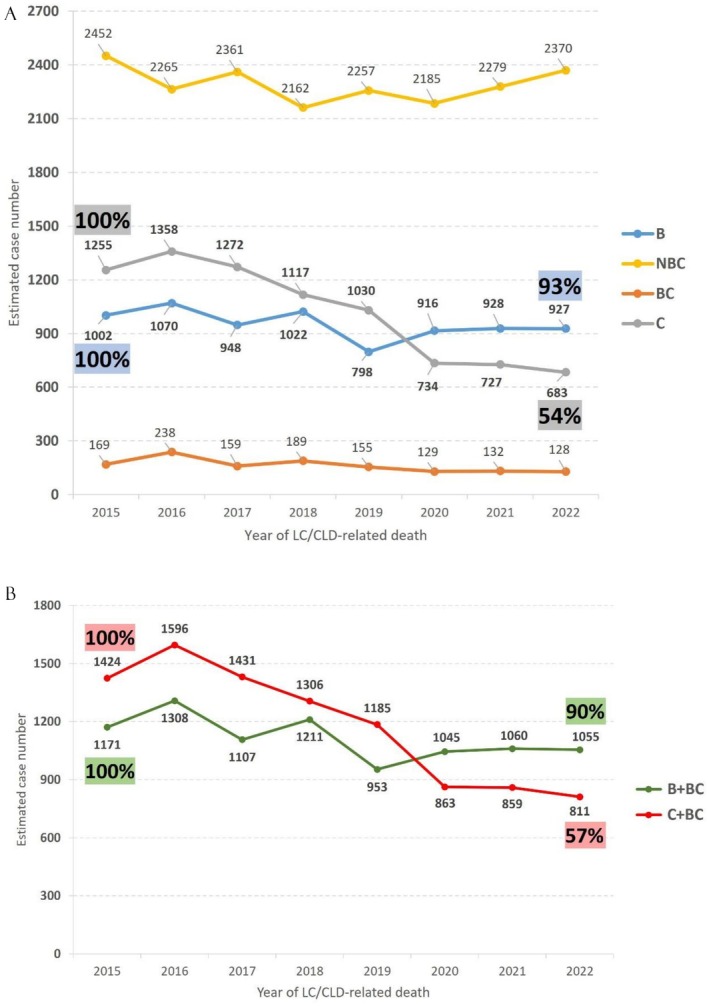
(A, B) The overall nationwide LC/CLD‐related deaths and secular trends were estimated based on the proportion of CGRD data. (A) Among anti‐HCV‐positive, HBsAg‐negative individuals, deaths declined by 46% from 2015 to 2022 (1255 to 683). (B) Among anti‐HCV‐positive individuals, regardless of HBsAg status, deaths declined by 43% from 2015 to 2022 (1424 to 811). B, HBsAg‐positive and anti‐HCV‐negative patients; BC, HBsAg‐ and anti‐HCV‐positive patients; C, HBsAg‐negative and anti‐HCV‐positive patients; LC/CLD, liver cirrhosis and chronic liver disease; NBC, HBsAg‐ and anti‐HCV‐negative patients.

After statistical adjustment, the CGRD data were extrapolated to estimate nationwide LC/CLD‐related deaths. The estimated number of anti‐HCV‐negative and HBsAg‐positive LC/CLD‐related deaths was 1002 in 2015 and 927 in 2022, with no significant change in trend. In contrast, the estimated number of anti‐HCV‐positive and HBsAg‐negative LC/CLD‐related deaths has decreased from 1255 in 2015 to 683 in 2022, representing a 46% reduction compared to 2015 (Figure 4A).

Including patients who tested positive for both anti‐HCV and HBsAg, the estimated number of LC/CLD‐related deaths among HBsAg‐positive individuals, regardless of anti‐HCV status, was 1171 in 2015 and 1055 in 2022, with no significant trend change. In contrast, the estimated number of LC/CLD‐related deaths among anti‐HCV‐positive individuals, regardless of HBsAg status, has declined from 1424 in 2015 to 811 in 2022, representing a 43% reduction (Figure 4B).

## 4 Discussion

Since the successful isolation and identification of HCV in 1989, the prevention and treatment of HCV infection have become major global research priorities [16]. Accumulating evidence indicates that HCV eradication significantly reduces the incidence of HCC, thereby contributing to a decline in HCC‐related mortality [22]. Notably, recent studies have revealed that the natural course of advanced chronic liver disease does not always progress in a stepwise fashion; in other words, HCV‐related deaths may occur even before the onset of hepatic decompensation or HCC [23]. These findings highlight the importance of evaluating HCV‐related mortality due to LC/CLD separately from HCC‐related deaths in order to capture the full spectrum of disease burden. However, distinguishing whether a patient's death is attributable to LC/CLD or HCC remains clinically challenging.

To meet the WHO target for HCV elimination by 2030, many countries have implemented national strategies for the prevention and treatment of chronic hepatitis C. In recent years, several epidemiological studies have been conducted to evaluate the effectiveness of these programs. A retrospective study conducted in three European countries revealed that over 75% of patients with chronic hepatitis C received treatment, with more than 95% achieving a sustained virological response [24]. In China, research has explored the prevalence of HCV infection across different populations [25, 26]. These studies generally follow WHO's 2023 recommendation to estimate national epidemiology using subnational sentinel surveillance systems. However, common limitations include relatively small sample sizes in comparison to national populations and geographically uneven study coverage.

Although our study also employed a subnational sentinel surveillance design, it stands out in its choice of sentinel center. The CGMH system spans Taiwan's most densely populated regions and leads the nation in both inpatient bed capacity and annual patient volume. It also houses the largest and most comprehensive electronic health record database in Taiwan, CGRD. Between 2015 and 2022, CGRD recorded 15 445 LC/CLD‐related deaths, representing 43.6% of all such deaths nationwide. Even after stringent exclusion criteria were applied, a substantial cohort of 5940 patients had complete HBsAg and anti‐HCV serology data—accounting for 16.8% of the national total. This extensive geographic coverage and large share of the national population strongly support the suitability and representativeness of CGMH as a sentinel center, addressing many of the limitations seen in previous studies.

Moreover, while some studies have investigated national trends in HCV‐related mortality, they often failed to exclude patients with pre‐existing HCC at baseline. This omission raises uncertainty regarding whether recorded deaths were truly attributable to HCV infection or HCC itself. To address this limitation, we adopted a dual‐database approach, integrating Taiwan's national mortality database with CGRD, and specifically excluded patients with confirmed HCC diagnoses. This methodology enhanced the accuracy of our estimates regarding HCV‐related mortality trends.

Given these methodological strengths, our findings highlight a significant reduction in HCV‐related LC/CLD mortality in Taiwan between 2015 and 2022. The 43% decline aligns with advancements in HCV treatment, particularly the widespread adoption of DAAs and national policies aimed at improving treatment accessibility. In January 2017, Taiwan's National Health Insurance (NHI) introduced conditional reimbursement for DAAs, prioritising patients with liver fibrosis stage F3 or higher [27, 28]. This policy substantially reduced treatment costs for HCV patients compared to the traditional ribavirin and interferon alpha (IFN‐α) regimen, providing more affordability, shorter duration and fewer adverse effects [29, 30]. In March 2020, NHI further expanded coverage, allowing all chronic HCV‐infected individuals to receive treatment regardless of fibrosis stage [31, 32]. Recognising disparities in the geographic distribution of hepatologists and to prevent patient loss during referral processes, NHI removed the specialty restriction for DAA prescriptions in October 2021, enabling a broader range of clinicians to provide HCV treatment. In addition to advancements in treatment policies, Taiwan Health Promotion Administration (HPA) has actively implemented nationwide HCV screening programs to enhance early detection. Since August 2011, the HPA has offered a one‐time free HCV screening for individuals aged 45 and above. In September 2020, the eligibility was further expanded to include individuals aged 45–79 [33]. These initiatives, combined with government‐led awareness campaigns and an increasing public health consciousness among the Taiwanese population, have significantly improved the identification of previously undiagnosed HCV cases. We believe that these efforts, along with improved treatment accessibility, have been the primary contributors to the substantial decline in HCV‐related mortality observed in recent years [10, 34].

In line with WHO recommendations, we reported crude mortality rates to evaluate reductions in mortality from hepatitis sequelae. While age‐adjusted rates are particularly valuable for international comparisons or for populations with markedly different demographic structures, our study was designed to assess longitudinal changes within the same national population over a relatively short 7‐year interval. This approach aimed to capture the real‐world decline in mortality in Taiwan following nationwide interventions.

We acknowledge, however, that the HCV‐infected population in Taiwan is gradually aging, which could potentially influence long‐term mortality trends. If the aging effect were predominant, it would be expected to contribute to an increase rather than a decrease in mortality. The observed decline therefore suggests that the impact of antiviral interventions outweighed demographic influences during the study period. Nevertheless, our findings should be interpreted with this limitation in mind, and future research incorporating age‐adjusted analyses and longer‐term outcomes, including hepatocellular carcinoma incidence, will be essential.

Another notable finding of this study is that more than half of the LC/CLD‐related deaths in recent years were among individuals negative for both HBsAg and anti‐HCV, with this trend being statistically significant (*p* < 0.001). Several studies have investigated the relationship between non‐B, non‐C liver disease and HCC, exploring the underlying etiologies and clinical characteristics [35, 36, 37, 38, 39, 40, 41, 42]. The high proportion of non‐B, non‐C cases in our cohort highlights the growing importance of alternative risk factors, such as metabolic dysfunction‐associated steatotic liver disease (MASLD) and alcohol‐related liver disease. To achieve a comprehensive reduction in all‐cause LC/CLD‐related mortality, future research on these etiologies is imperative.

Overall, this study makes a significant contribution to the understanding of HCV‐related mortality by addressing key surveillance gaps in non‐cancerous liver disease. By integrating Taiwan's national mortality database with the CGRD—the country's largest multi‐institutional electronic health records repository—we provide robust, population‐based estimates of HCV‐related LC/CLD mortality. These findings not only clarify the epidemiological burden of HCV‐related liver disease in Taiwan but also offer a scalable analytic framework for other countries with limited national serological data. In line with WHO recommendations for using subnational sentinel data in resource‐constrained settings, our approach demonstrates the feasibility and utility of leveraging institutional databases for nationwide disease surveillance and program evaluation. Moreover, our findings demonstrate a marked decline in HCV‐related LC/CLD mortality in recent years, reflecting the effectiveness of Taiwan's national screening and treatment policies. These outcomes may serve as valuable references for other countries pursuing similar viral hepatitis elimination goals. As such, the implications of this study extend beyond Taiwan and contribute valuable evidence to the global discourse on viral hepatitis elimination.

This study had two primary limitations. First, although the CGMH institutions cover northern, central, southern and western Taiwan, it lacks facilities in the less populated eastern region. While the CGRD remains the largest and most comprehensive medical database in Taiwan, this geographic gap may introduce selection bias and limit the generalisability of our findings. To address this, we plan to collaborate with another hospital system that includes medical centers in eastern Taiwan. Future data integration with this institution may help mitigate regional disparities. Second, the classification of underlying etiologies in this study was primarily based on the presence of HBsAg and anti‐HCV serological markers, with limited information on other important causes such as MASLD and alcohol‐related liver disease. These conditions may coexist with HCV and independently contribute to liver‐related mortality, potentially leading to an overestimation of HCV‐attributable deaths. As these factors cannot be readily extracted from the CGRD, future studies will incorporate manual chart reviews and potentially natural language processing techniques to extract relevant keywords and clinical data from large volumes of unstructured records, thereby improving etiological classification and mortality attribution [43, 44, 45].

In summary, this study revealed a steady decline in nationwide mortality associated with LC/CLD in recent years, with a reduction of 15.8%. Additionally, analysis of CGRD data demonstrated a significant 36.2% decrease in the proportion of cases testing positive for anti‐HCV. Given that CGMH serves as a representative sentinel center in Taiwan, we estimate that from 2015 to 2022, the nationwide mortality related to anti‐HCV‐associated LC/CLD has declined by 43%.

## Author Contributions

**Cheng‐Yeh Yang:** conceptualisation, data analysis, interpretation of results, literature review, manuscript drafting. **Chih‐Yun Lin:** data acquisition, statistical analysis, interpretation of results. **Jing‐Houng Wang:** clinical input, validation. **Chun‐Yen Lin:** clinical input, quality control. **Rong‐Nan Chien:** clinical input, quality control. **Sheng‐Nan Lu:** supervision, study design, critical revision of the manuscript for important intellectual content.

## Conflicts of Interest

The authors declare no conflicts of interest.

## Data Availability

The data that support the findings of this study are available on request from the corresponding author. The data are not publicly available due to privacy or ethical restrictions.
